# ENHANCING AGE-FRIENDLY CARE IN VALUE-BASED HEALTH SYSTEMS: CMS DELIRIUM ASSESSMENT AND POLICY STRATEGY

**DOI:** 10.1093/geroni/igae098.4261

**Published:** 2024-12-31

**Authors:** Tara McMullen, Shari Ling, Lenise Cummings-Vaughn

**Affiliations:** Centers for Medicare & Medicaid Services, Baltimore, Maryland, United States; Centers for Medicare & Medicaid Services, Baltimore, Maryland, United States; Centers for Medicare & Medicaid Services, Baltimore, Maryland, United States

## Abstract

Delirium is a clinical syndrome characterized by alteration in consciousness and cognitive function, frequently observed in older adults. This condition is associated with a risk of mortality and can lead to severe and persistent functional impairments in survivors. The incidence of delirium is notably high, affecting up to 80% of older adults in intensive care units (ICUs) and up to 35% of those with general inpatient admissions. Routine and standardized cognitive assessments are essential for optimizing patient management and implementing delirium prevention strategies aimed at mitigating risk factors. To enhance person-centered care in delirium management, it is crucial to adopt age-friendly care frameworks that emphasize evidence-based approaches to assessment and management. In this presentation, Federal officials from the Centers for Medicare & Medicaid Services (CMS) will address the ongoing clinical and policy initiatives aimed at developing a comprehensive strategy for the screening and assessment of delirium, integrated within the framework of high-quality, age-friendly healthcare. This presentation will explore the essential attributes that health systems must possess to effectively translate age-friendly principles into improved health outcomes for patients with delirium. Officials will also discuss the development of age-friendly measures that address the specific needs of older adults to enhance both individual outcomes and population-level health. Additionally, the discussion will focus on policy levers that should be considered to promote age-friendly, value-based care, thereby improving the screening and prevention of delirium.

